# Giant Bulk Photovoltaic Effect in Two‐Dimensional Topological Ferroelectric Semimetal

**DOI:** 10.1002/advs.75863

**Published:** 2026-06-12

**Authors:** Jianhua Wang, Xia Cheng, Shibo Fang, Tao Zhu, Xiaodong Zhou, Shifeng Qian, Tie Yang, Wenhong Wang, Yee Sin Ang

**Affiliations:** ^1^ School of Materials Science and Engineering Tiangong University Tianjin China; ^2^ Science, Mathematics and Technology (SMT) Cluster Singapore University of Technology and Design Singapore Singapore; ^3^ School of Physical Science and Technology Southwest University Chongqing China; ^4^ Institute of Quantum Materials and Devices, School of Electronics and Information Engineering Tiangong University Tianjin China; ^5^ Anhui Province Key Laboratory for Control and Applications of Optoelectronic Information Materials, Department of Physics Anhui Normal University Wuhu Anhui China

**Keywords:** bulk photovoltaic effect, ferroelectric metal, topological semimetal, weyl points

## Abstract

Ferroelectric (FE) metals have attracted growing interest because they combine ferroelectricity with metallic behavior and enable polarization‐controlled band topology near the Fermi level. However, the number of known ferroelectric metals is still limited. Here, we employ first‐principles calculations and symmetry analysis to identify three two‐dimensional (2D) ferroelectric metal candidates—XO monolayers (X = Nb, Ta, Rh)—featuring considerable out‐of‐plane polarization and moderate switching barriers. Notably, the polarization of RhO reaches 5.68 pC/m, significantly exceeding those of most reported 2D FE metals. The XO monolayers are also ferroelectric topological semimetals, hosting symmetry‐protected Weyl points along the S‐Y path enforced by the mirror symmetry *M_y_*, with nontrivial edge states emerging at the projected Weyl points. We also demonstrate an intrinsically large bulk photovoltaic effect (BPVE), arising from the nontrivial band topology and the broken inversion symmetry in the ferroelectrics. Such BPVE responses can be further enhanced through charge doping and strain engineering. Our work establishes XO monolayers as a versatile 2D platform that integrates switchable ferroelectricity with topological semimetal behavior and a giant BPVE, enabling multifunctional nanoelectronic and optoelectronic applications.

## Graphical abstract

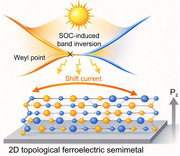

## 1 Introduction

Over the past few decades, ferroelectric (FE) materials have attracted significant attention due to their unique physical properties [1, 2, 3, 4]. Their capability to switch spontaneous polarization in response to an external electric field positions them as promising candidates for applications in information storage, nano‐spintronic devices, and sensors [5, 6, 7, 8]. Traditionally, however, the use of FE materials was largely restricted to insulators and semiconductors, as the presence of free electrons in metals often screens the internal electric fields necessary for sustaining spontaneous polarization. This paradigm began to shift when Anderson and Blount [9] theoretically proposed that metals could undergo symmetry‐breaking structural transitions into polar phases. Following this theoretical advancement, Shi et al. [10] experimentally demonstrated a continuous transition from a centrosymmetric to a polar structure in bulk ${\mathrm{LiOsO}}_{3}$ while maintaining metallic conductivity. This breakthrough marked a significant milestone, expanding the classification of FE materials to encompass metals alongside traditional insulators and semiconductors.

In two‐dimensional (2D) systems, metallic and FE states can coexist. While conductive electrons in 2D materials are primarily confined to in‐plane motion, external electric fields can still facilitate switching of out‐of‐plane polarization. Several 2D FE metals have been theoretically proposed, including ${\mathrm{LiOsO}}_{3}$ [11], CrN [12], ${\mathrm{CrB}}_{2}$ [12], ${\mathrm{Bi}}_{5}$ ${\mathrm{Ti}}_{5}$ $O_{17}$ [13], $M_{I}$ $M_{\mathrm{II}}$ $P_{2}$ $X_{6}$ [14], ${\mathrm{Os}}_{2}$ ${\mathrm{Se}}_{3}$ [15], ${\mathrm{PtBi}}_{2}$ [16], and slid bilayer ${\mathrm{Fe}}_{5}$ ${\mathrm{GeTe}}_{2}$ [17]. However, experimental validation of these 2D FE metals presents significant challenges; to date, only multilayer 1T'‐${\mathrm{WTe}}_{2}$ has been experimentally confirmed [18]. A major obstacle in this field is the limited number of viable candidate materials. Consequently, the search for and systematic screening of potential 2D FE metals has become an urgent research priority.

The bulk photovoltaic effect (BPVE) arises from the shift current generated under uniform illumination, attracting significant attention for its potential to directly convert light into electricity [19, 20, 21, 22]. A critical requirement for the BPVE is the absence of inversion symmetry (P), making FE materials—characterized by spontaneous polarization and broken inversion symmetry—ideal platforms for studying this phenomenon [23, 24]. Since the shift current is central to the BPVE [20], it has been extensively measured in a variety of FE materials to characterize their photovoltaic responses [7, 23, 24]. A primary focus within this field is the pursuit of large shift currents. In non‐magnetic systems, nontrivial band topology can significantly enhance optical transitions, thereby leading to substantial shift currents [25]. This has spurred interest in topological semimetals, where shift currents exhibit low‐frequency divergence at Dirac points, Weyl points, and nodal lines [25, 26]. Notably, type‐II Dirac and Weyl points demonstrate particularly pronounced low‐frequency divergence due to their strong anisotropic properties. While the BPVE in type‐II Dirac/Weyl systems [26, 27, 28] as well as the shift currents in 2D FE metals [16] (e.g., ${\mathrm{PtBi}}_{2}$) and FE semiconductors [29] (e.g., single‐layer monochalcogenides) have been reported, 2D FE semimetals—promising candidates for achieving large shift currents—remain largely unexplored.

In this work, we identify three candidate FE metals, denoted as XO (where X = Nb, Rh, Ta), using the Computational 2D Materials Database (C2DB) [30] through first‐principles calculations and theoretical analysis. These XO monolayers belong to the pm11 layer group and demonstrate dynamic stability in their ground‐state configurations. They exhibit pronounced out‐of‐plane spontaneous polarization and low energy barriers for polarization switching, thereby confirming their potential as 2D FE metals. From an electronic perspective, the monolayers display metallic behavior and feature type‐II Weyl points near the Fermi level, with discernible nontrivial edge states linked to the projected Weyl points. When spin‐orbit coupling (SOC) is incorporated, small energy gaps are opened at the Weyl points, accompanied by band inversion in the adjacent regions. Importantly, the nontrivial band topology of these materials facilitates a significantly enhanced shift current, which can be further effectively modulated through external strain or by adjusting the position of the Fermi level.

## 2 Results and Discussion

### 2.1 Crystal Structure and Ferroelectric Metal

**FIGURE 1:**
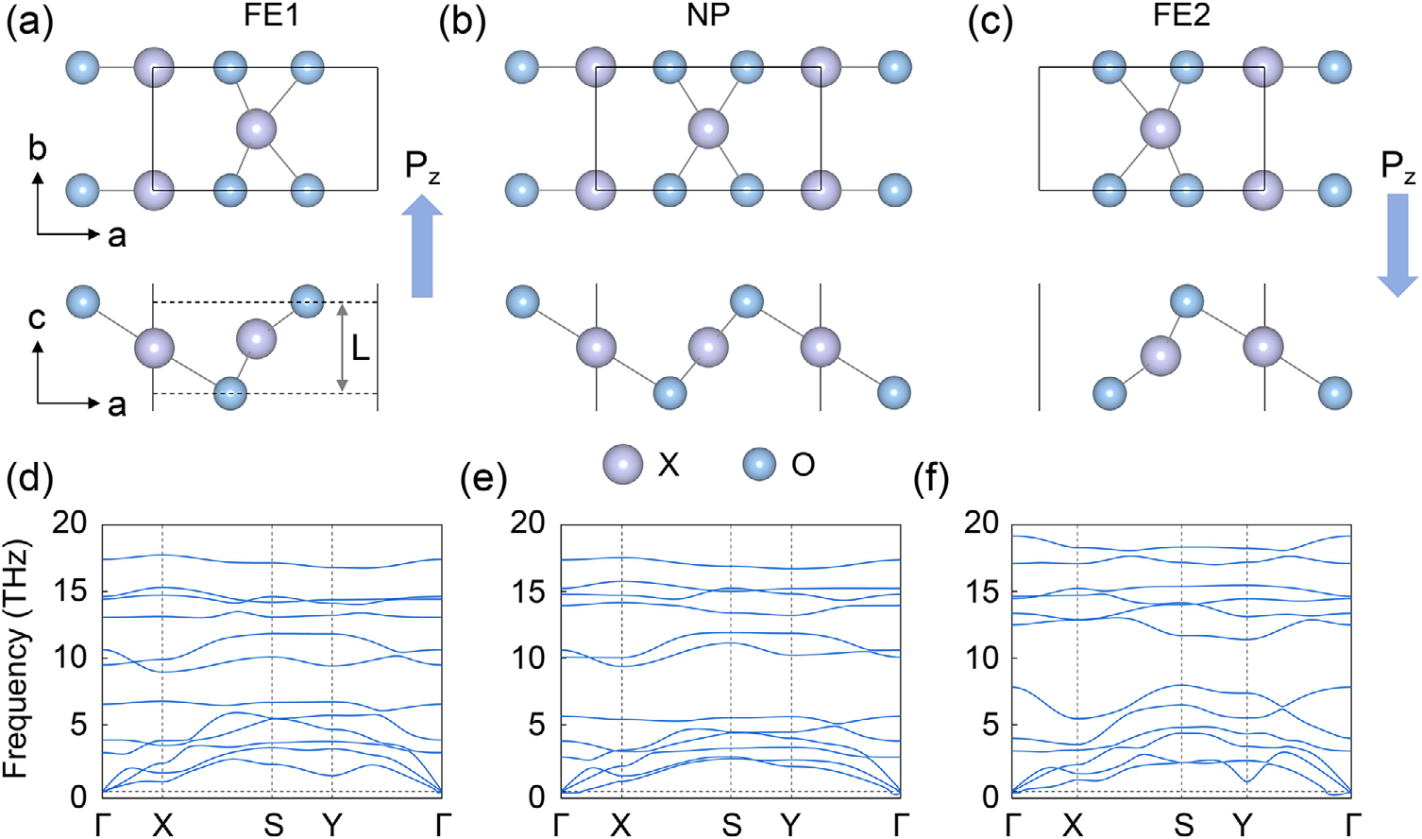
(a–c) Crystal structures of the XO (X = Nb, Ta, Rh) monolayers in the ferroelectric FE1, nonpolar (NP), and ferroelectric FE2 states, respectively. Purple and blue spheres denote the metal (X) and oxygen atoms, respectively; L indicates the effective monolayer thickness. Phonon dispersions of ferroelectric XO monolayers. (d) NbO, (e) TaO, and (f) RhO.

From the C2DB database, we have identified three monolayer materials—XO, where X = Nb, Ta, and Rh—that exhibit metallic electronic structures and out‐of‐plane polarization. These monolayers belong to the pm11 (No. 11) layer group and possess an in‐plane mirror symmetry $M_{y}$. Each unit cell contains two X atoms and two oxygen atoms, with the oxygen atoms occupying the Wyckoff position 1a (x, 0, z) and the X atoms occupying Wyckoff positions 1a and 1b (x, 1/2, z), as illustrated in Figure 1a. Following full structural optimization, the in‐plane lattice constants were determined as follows: a = 5.29 Å and b = 2.89 Å for NbO; a = 4.73 Å and b = 2.97 Å for RhO; and a = 5.23 Å and b = 2.91 Å for TaO. Phonon dispersion calculations confirm the dynamic stability of the ground‐state structures of these XO materials, as shown in Figure 1d–f. The absence of imaginary frequencies across the entire Brillouin zone (BZ) indicates that these monolayers can exist stably, making them promising candidates for experimental synthesis.

**FIGURE 2:**
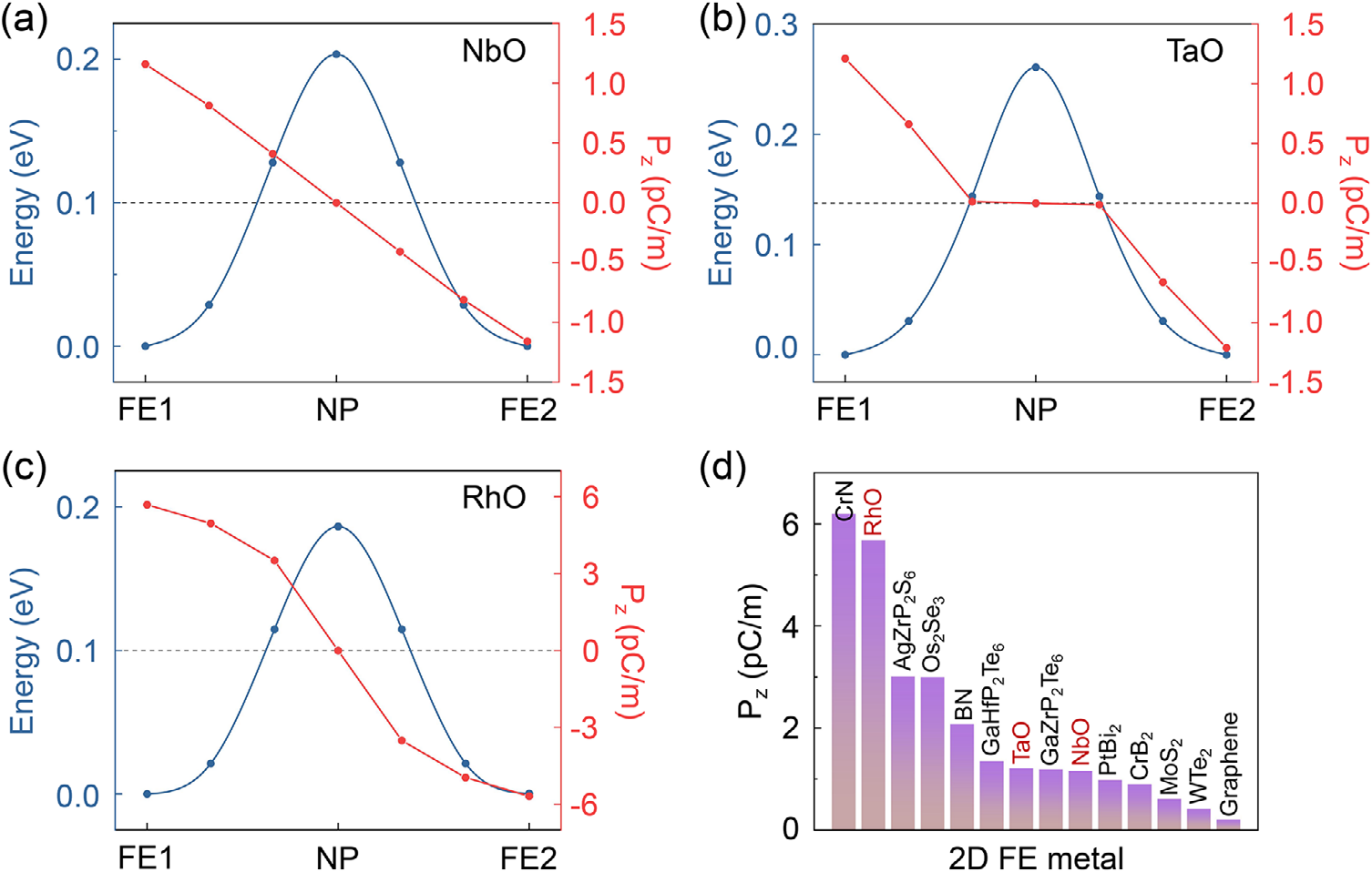
(a–c) Calculated energy barriers and evolution of the out‐of‐plane polarization ($P_{z}$) along the FE1 to FE2 transition path for NbO, TaO, and RhO, respectively. (d) Comparison of the out‐of‐plane polarization between the XO monolayers and other typical 2D FE metals.

The XO monolayers crystallize in point group m, which is one of the ten polar point groups. The absence of both inversion symmetry and out‐of‐plane mirror symmetry $M_{z}$ results in a spontaneous out‐of‐plane polarization $P_{z}$. As illustrated in Figure 1a,c, we identify two FE metallic states (FE1 and FE2) characterized by degenerate ground‐state energies but opposite polarization values of ± $P_{z}$. The out‐of‐plane polarization for a 2D material is defined as [16]

(1) $$P_{z}=\frac{1}{S}\int Z\left(P_{ions}+P_{valence}\right)d^{3}r,$$

where S is the unit‐cell area, and $P_{ions}$ and $P_{valence}$ denote the charge densities of ionic cores and valence electrons along the out‐of‐plane direction, respectively. As shown in Figure 2a– c, the calculated $P_{z}$ values in their FE states are 1.16 pC/m for NbO, 1.21 pC/m for TaO, and 5.68 pC/m for RhO in their FE states. Notably, the polarization of RhO significantly exceeds that of other reported 2D FE metals [12, 14, 15, 16, 18, 31, 32, 33] (Figure 2d), and is only slightly lower than that of CrN, which exhibits a polarization of 6.20 pC/m.

In the presence of an external electric field, the transition between the two FE states of 2D FE metals involves a continuous structural evolution, with a centrosymmetric nonpolar (NP) phase emerging as an intermediate metastable state, as illustrated in Figure 1b. The polarization switching pathways were calculated using the solid‐state nudged elastic band (SS‐NEB) method [34], with results depicted in Figure 2a–c. During the transition between the stable FE phases, the polarization undergoes a sign reversal, with the energy barrier peaking at the centrosymmetric NP phase. The calculated switching barriers are 0.20 eV per unit cell for NbO, 0.26 eV per unit cell for TaO, and 0.19 eV per unit cell for RhO. These values are comparable to those reported for other FE metals, such as ${\mathrm{Os}}_{2}$ ${\mathrm{Se}}_{3}$ (0.22 eV/unit cell) [15], ${\mathrm{PtBi}}_{2}$ (0.378 eV/unit cell) [16], and Cr(h‐fpyz)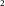 (0.23 eV per h‐fpyz) [35]. Both the moderate switching barriers and the substantial polarization values underscore the potential of XO monolayers as intrinsic FE metals.

### 2.2 Band Topology

**FIGURE 3:**
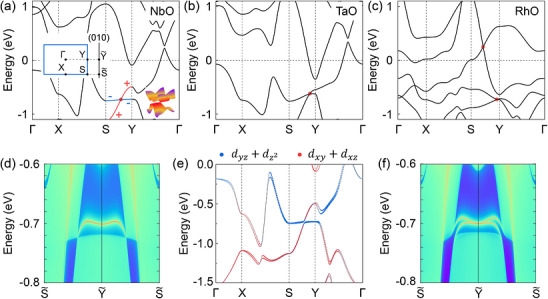
(a–c) Electronic band structures of NbO, TaO, and RhO, respectively. The insets in panel (a) depict the corresponding 2D BZ, its projection along the (010) direction, and the band dispersion near the Weyl point in the $k_{x}$‐$k_{y}$ plane along $k_{z}$. (d) Edge states of NbO without SOC. (e) Orbital‐projected band structure of NbO with SOC included. (f) Corresponding edge states in the presence of SOC.

The electronic band structures of the XO monolayers were calculated along the high‐symmetry path Γ‐X‐S‐Y‐Γ, with the corresponding 2D BZ shown in the inset of Figure 3a. All three monolayers exhibit metallic behavior, as presented in Figure 3a–c. Weyl points, marked by red dots, are identified along the S‐Y path. Specifically, in NbO, a Weyl point located at approximately ‐0.73 eV along the S‐Y direction reveals a characteristic band tilting in the $k_{z}$ direction within the $k_{x}$‐$k_{y}$ plane (inset of Figure 3a), confirming its type‐II nature. Similarly, TaO hosts a type‐II Weyl point situated within ±1 eV of the Fermi level. In contrast, RhO presents two type‐I Weyl points which are characterized by non‐tilted band crossings, as seen in Figure 3b,c.

Considering the identical symmetry operations present in all XO monolayers, we select NbO as a representative system to investigate their topological characteristics (see the Supporting Information for TaO and RhO). Theoretical analysis reveals that the linear band crossings along the S‐Y path on the BZ boundary are protected by the mirror symmetry $M_{y}$. The calculated $M_{y}$ eigenvalues of the two bands forming the Weyl point are opposite, confirming that this crossing is indeed symmetry‐protected by the $M_{y}$ mirror plane. To examine the bulk‐boundary correspondence associated with the Weyl points, we compute the topologically protected edge states of NbO along the (010) direction. As shown in Figure 3d, the edge band structure along the $\tilde{S}$‐$\tilde{Y}$‐$\tilde{S}$ path clearly exhibits nontrivial edge states terminating at the projected Weyl point. This correspondence between bulk and boundary states corroborates the nontrivial topology of the Weyl point in NbO. The well‐defined edge states further provide a clear signature for future experimental verification.

When SOC is incorporated, the Weyl points become gapped, resulting in a small but finite bandgap. Band inversion occurs around these gapped crossings. To verify this, we computed the orbital‐projected band structure of NbO, emphasizing the contributions from the Nb d‐orbitals, as presented in Figure 3e. The results clearly indicate an inversion between Nb d‐orbital subsets—specifically between ($d_{yz}$, $d_{z^{2}}$) and ($d_{xy}$, $d_{xz}$)—in the vicinity of the original Weyl point location. Similar behavior is also observed in TaO and RhO, which is anticipated to enhance the BPVE in these monolayers. Furthermore, the edge spectrum depicted in Figure 3f reveals gapless edge states that terminate at the projections of the gapped Weyl points on either side of the bandgap. Experimentally, the band structure of the Weyl points and the associated nontrivial edge states can be probed using angle‐resolved photoemission spectroscopy (ARPES).

### 2.3 Bulk Photovoltaic Effect

The shift current, which emerges in the ferroelectric state under uniform illumination, is described by a third‐rank tensor $\sigma^{abc}\left(0;\omega ,-\omega \right)$. The current density is given by

(2) $$J_{shift}^{a}\left(\omega \right)=2\Sigma_{bc}\sigma^{abc}\left(0;\omega ;-\omega \right)E^{b}\left(\omega \right)E^{c}\left(-\omega \right),$$

where a, b, c denote Cartesian indices, and E(ω) is the Fourier component of the electric field at angular frequency ω. The shift‐current tensor is expressed as [20]

(3) $$\begin{array}{ccc} & & \sigma^{abc}\left(0;\omega ;-\omega \right)\\ & & \;=-\frac{i\pi e^{3}}{2\hbar^{2}}\int \frac{dk}{8\pi^{3}}\Sigma_{nm}f_{nm}\left(r_{mn}^{b}r_{nm;a}^{c}+r_{mn}^{c}r_{nm;a}^{b}\right)\delta \left(\omega_{mn}-\omega \right). \end{array}$$

Here, $f_{nm}=f_{n}-f_{m}$ is the difference in Fermi‐Dirac occupation factors, $\hbar \omega_{nm}=\hbar \omega_{n}-\hbar \omega_{m}$ is the energy difference between bands n and m, and $r_{mn}^{a}$ is the velocity matrix element. Here, $r_{mn;b}^{a}=\partial r_{mn}^{a}/\partial k^{b}-i\left({A_{nn}}^{b}-A_{mm}^{b}\right)r_{nm}^{a}$ denotes the generalized derivative, where $A_{nm}^{a}$ is the Berry connection. For a 2D system with slab thickness H and effective thickness L, the shift conductivity analogous to the three‐dimensional (3D) case can be written as [36]

(4) $$\sigma^{abc}\left(0;\omega ;-\omega \right)=\frac{H}{L}\sigma_{slab}^{abc}.$$

**FIGURE 4:**
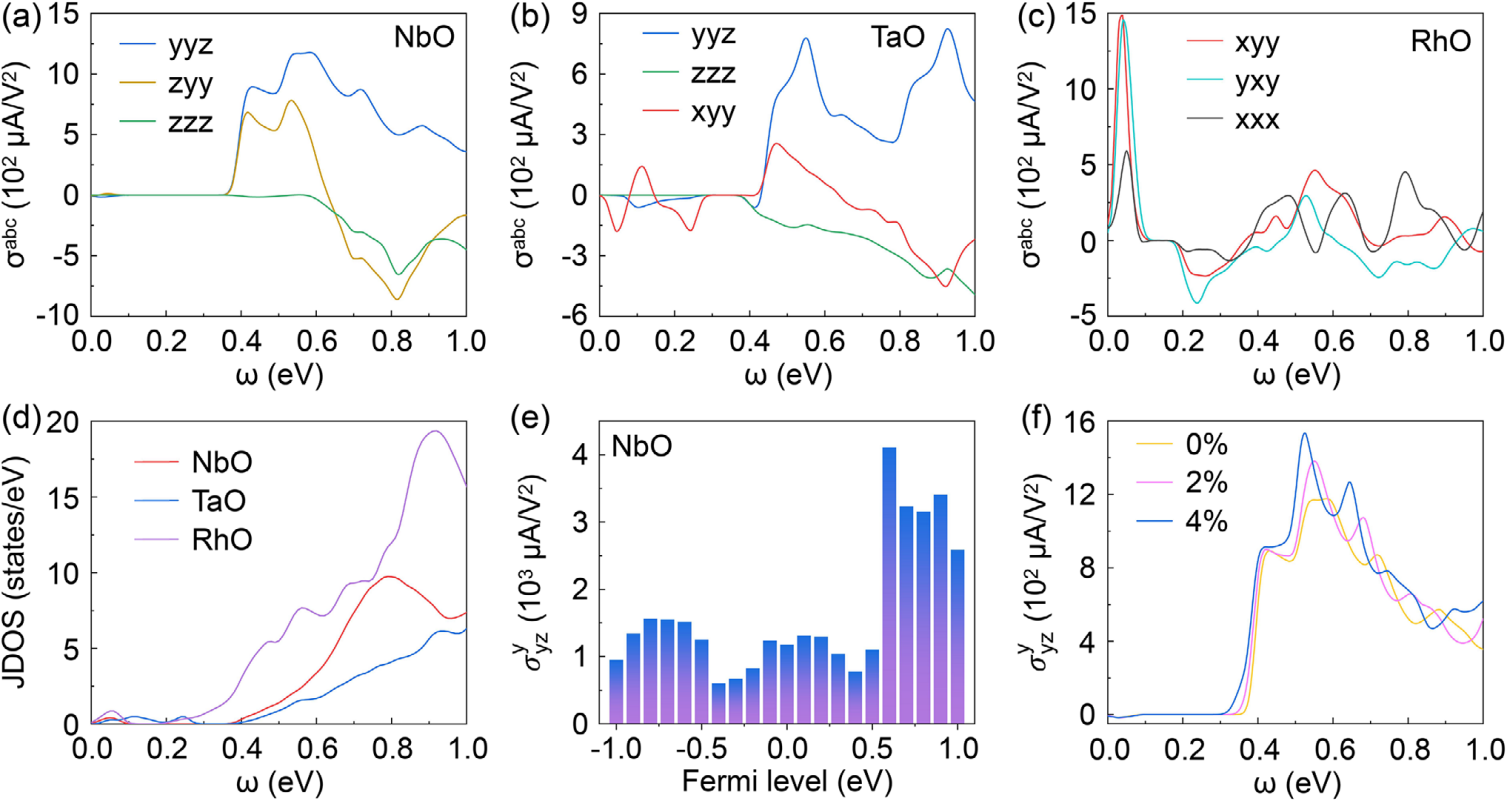
(a–c) Shift current spectra of NbO, TaO, and RhO, respectively, including SOC. (d) Joint density of states (JDOS) for the three XO monolayers. (e) Shift current conductivity $\sigma_{yz}^{y}$ of NbO as a function of Fermi‐level position. (f) Strain dependence of the shift current conductivity $\sigma_{yz}^{y}$ in NbO under biaxial tensile strain.

We utilize the shift current to assess the strength of the BPVE in the FE states of the proposed XO monolayers. The third‐rank shift‐current tensor $\sigma^{abc}\left(0;\omega ,-\omega \right)$ can be decomposed into 18 independent tensor components. In the XO monolayers, the presence of mirror symmetry $M_{y}$ necessitates three components—$\sigma_{xx}^{y}$, $\sigma_{yy}^{y}$, $\sigma_{xy}^{x}$—to vanish. However, symmetry alone does not dictate the magnitude of the remaining components. Therefore, we identify the three largest shift‐current components for each material. As shown in Figure 4a– c, the dominant components for NbO are $\sigma_{yz}^{y}$, $\sigma_{yy}^{z}$ and $\sigma_{zz}^{z}$. The $\sigma_{yz}^{y}$ component reaches a maximum of approximately 1176 μA/$V^{2}$ at around 0.58 eV. For TaO, the largest components are $\sigma_{yz}^{y}$, $\sigma_{zz}^{z}$ and $\sigma_{yy}^{x}$, with $\sigma_{yz}^{y}$ peaking near 820 μA/$V^{2}$ at 0.93 eV. In RhO, the most prominent components are $\sigma_{yy}^{x}$, $\sigma_{xy}^{y}$ and $\sigma_{xx}^{x}$, where $\sigma_{yy}^{x}$ attains a maximum of 1479 μA/$V^{2}$ at approximately 0.04 eV. These divergent profiles are likely a consequence of the intrinsic anisotropy exhibited by each material. The enhancement of the shift current can be linked to the joint density of states (JDOS), defined as [25]

(5) $$D_{joint}\left(\omega \right)=\frac{\Omega }{\hbar }\int \frac{d^{3}k}{{\left(2\pi \right)}^{3}}\Sigma_{n,m}f_{nm}\delta \left(\omega_{mn}-\omega \right),$$

where Ω is the unit‐cell volume. The calculated JDOS for the XO monolayers is presented in Figure 4d. The peak structures in the shift‐current tensor components closely align with the features observed in the JDOS. Notably, the shift‐current magnitudes obtained are significantly larger than those reported for other 2D FE materials, such as ${\mathrm{PtBi}}_{2}$ (400 μA/$V^{2}$) [16] and single‐layer monochalcogenides (100 μA/$V^{2}$) [29].

The shift current is influenced not only by the JDOS but also by the position of the Fermi level. Using NbO as an illustrative example, Figure 4e shows how the maximum of the shift current component $\sigma_{yz}^{y}$ evolves as the Fermi level is varied between –1 eV and +1 eV. The shift current reaches a peak value of approximately 4100 μA/$V^{2}$ when the Fermi level is tuned to around 0.6 eV, a condition that can be achieved experimentally through electron doping (see the Supporting Information). Additionally, we investigate the impact of strain on the shift current. As presented in Figure 4f, applying biaxial tensile strain of 2% and 4% enhances the shift current response. It is noteworthy that the two FE states can be switched using an external electric field, which simultaneously reverses the sign of the corresponding shift current. These findings demonstrate that the magnitude of the shift current can be effectively tuned by doping and strain, while its direction can be controlled by modulating the FE polarization with an external electric field.

## 3 Conclusions

In summary, we present three 2D FE metals, the XO monolayers (where X = Nb, Ta, Rh). These materials demonstrate dynamic stability and exhibit moderate energy barriers for polarization switching, accompanied by significant out‐of‐plane polarization ($P_{z}$). Their electronic structures feature Weyl points along the Y‐S path, which are protected by mirror symmetry. When SOC is incorporated, these Weyl points lead to band inversion and the opening of a bandgap. The noncentrosymmetric crystal structure provides the essential conditions for the BPVE, while the nontrivial band topology considerably enhances the shift current. Under an external electric field, both the FE polarization and the direction of the shift current can be effectively reversed. We further demonstrate that the magnitude of the shift current can be finely tuned through carrier doping (either electrons or holes) and by applying external strain. Notably, NbO monolayer is composed of elements posing low risk to human and envrionment without supply risk, thus suggesting its potential in sustainable green electronics [37]. Our findings not only broaden the landscape of 2D FE metals but also establishes XO monolayers as a promising platform for investigating and engineering the BPVE, potentially paving the way for future electronic and optoelectronic devices.

## 4 Calculation Method

First‐principles calculations were performed within the framework of density functional theory (DFT) using the Vienna ab initio Simulation Package (VASP) [38, 39]. The exchange‐correlation functional was treated with the Perdew–Burke–Ernzerhof (PBE) form of the generalized gradient approximation (GGA) [40]. The projector augmented‐wave (PAW) [41] method was utilized with a plane‐wave cutoff energy of 500 eV. During structural optimizations, both the lattice parameters and atomic coordinates were fully relaxed until electronic convergence thresholds of 10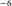 eV for the total energy and 0.001 eV/Å for the atomic forces were reached. A Monkhorst–Pack k‐mesh [42] of 7×11×1 was employed for BZ sampling during structural optimization and electronic structure calculations. To mitigate spurious interactions between periodic images, a vacuum layer exceeding 15 Å was introduced. Phonon dispersions were obtained via density functional perturbation theory (DFPT) as implemented in the PHONOPY package [43]. The irreducible representations of the electronic bands were analyzed using the IRVSP code [44]. A tight‐binding model was constructed from the DFT results using the WANNIER90 package [45] and subsequently employed to compute the shift current. Edge states were calculated using the WANNIERTOOLS package [46]. The nonlinear photoconductivity (shift current) was evaluated within the Wannier tight‐binding framework by integrating over the BZ with a dense k‐mesh of 1500×1500×1. Details on the comparison between DFT and Wannier model band structures of XO monolayers, along with the convergence of the peak shift current with k‐mesh density, are provided in the Supporting Information.

## Conflicts of Interest

The authors declare no conflicts of interest.

## Supporting information


**Supporting File**: advs75863‐sup‐0001‐SuppMat.pdf.

## Data Availability

The data that support the findings of this study are available from the corresponding author upon reasonable request.
