# Supplementary material for: Giant Bulk Photovoltaic Effect in Two‐Dimensional Topological Ferroelectric Semimetal
Source: Adv Sci (Weinh). 2026 Jun 12:e75863. Online ahead of print. doi: 10.1002/advs.75863 (PMC13336021; doi:10.1002/advs.75863)
Supplement: Supplementary file 1 — Supporting File: advs75863‐sup‐0001‐SuppMat.pdf. [file ADVS-9999-e75863-s001.pdf]

Supporting Information for "Giant Bulk Photovoltaic Effect in Two-Dimensional Topological Ferroelectric Semimetal"

Jianhua Wang, Xia Cheng, Shibo Fang, Tao Zhu, Xiaodong Zhou, Shifeng Qian, Tie Yang, Wenhong Wang*, Yee Sin Ang**

J. Wang, W. Wang
School of Materials Science and Engineering, Tiangong University, Tianjin 300387, China
Email: wenhongwang@tiangong.edu.cn
J. Wang, S. Fang, Y. S. Ang
Science, Mathematics and Technology (SMT) Cluster, Singapore University of Technology and Design, Singapore 487372, Singapore
Email: yeesin_ang@sutd.edu.sg
X. Cheng, T. Yang
School of Physical Science and Technology, Southwest University, Chongqing 400715, China.
E-mail: yangtie@swu.edu.cn
T. Zhu, X. Zhou, W. Wang
Institute of Quantum Materials and Devices, School of Electronics and Information Engineering, Tiangong University, Tianjin, 300387, China.
S. Qian
Anhui Province Key Laboratory for Control and Applications of Optoelectronic Information Materials, Department of Physics, Anhui Normal University, Wuhu Anhui 241000, China.

We construct Wannier-based tight-binding (TB) models for NbO, TaO, and RhO by selecting the s, p, and d orbitals of Nb, Ta, and Rh, respectively, together with the s and p orbitals of O. As shown in **Figure S1**, the band structures from the Wannier functions are in excellent agreement with the DFT results. In the absence of SOC, the edge band structure of TaO along the (010) direction clearly exhibits nontrivial edge states terminating at the Weyl points, as shown in **Figure S2**a. When SOC is included, the Weyl points are destroyed and a band gap opens. Near the original Weyl points, band inversion occurs between the (d_{yz}, d_{z^2}) and (d_{xy}, d_{xz}) orbitals of Ta, as illustrated in **Figure S2**b. The resulting gap is bridged by the edge states, as shown in **Figure S2**c. NbO and TaO exhibit similar topological characteristics. In contrast, for RhO, the edge states along the (010) direction are obscured by bulk bands, and no clearly identifiable nontrivial edge states can be identified (**Figure S2**d,f). Notably, for RhO, the two Weyl points near the Fermi level along the S-Y path exhibit SOC-induced band inversions between the Rh d_{xy} and d_{z^2} orbitals, as well as between the Rh d_{z^2} and d_{xz} orbitals (**Figure S2**e).

To assess the convergence of the peak shift current with respect to k-mesh density in XO monolayers, we take the σ^{y}_{yz} component for NbO and TaO, and the σ^{x}_{yy} component for RhO under SOC as representative examples. As shown in **Figure S3**, the peak shift current shows negligible variation beyond a k-mesh density of 1500×1500×1, indicating good convergence. **Figure S4** shows the shift current spectra of XO monolayers in the absence of SOC. The maximum values are found to be 652 μA/V^2 for NbO, 707 μA/V^2 for TaO, and 325 μA/V^2 for RhO. These results indicate that SOC-induced band inversion can effectively enhance the shift current. It is worth noting that the Fermi level can be tuned via electron or hole doping. As shown in **Figure S5**, upon electron doping of 1.26 electrons in NbO, the Fermi level shifts upward by 0.6 eV relative to the undoped case, corresponding to a carrier concentration of 8.2×10^{14} cm^{-2}.

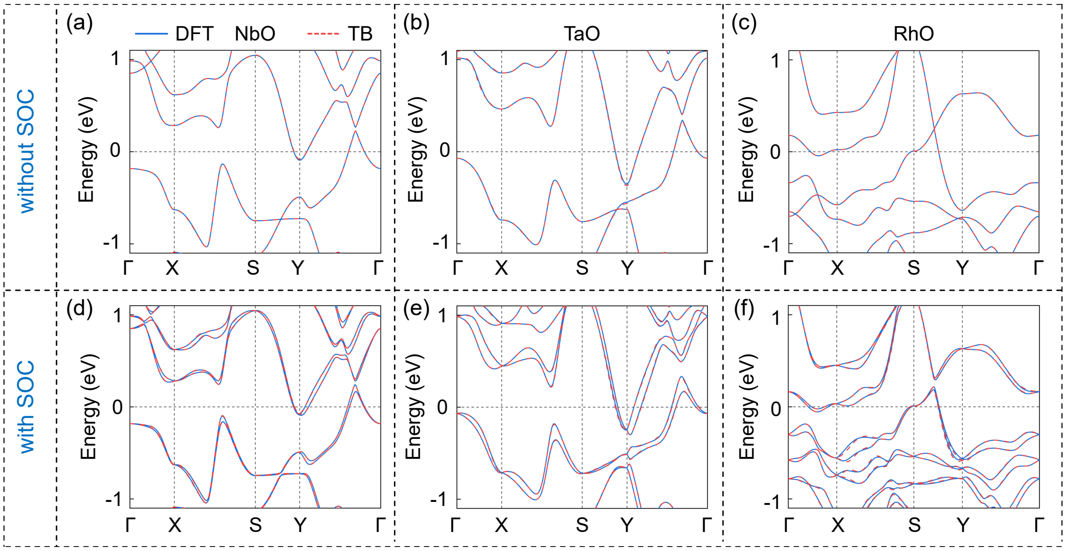

Figure S1: Comparison of density functional theory (DFT) and Wannier-model band structures for NbO [(a), (d)], TaO [(b), (e)], and RhO [(c), (f)], shown without and with spin-orbit coupling (SOC).

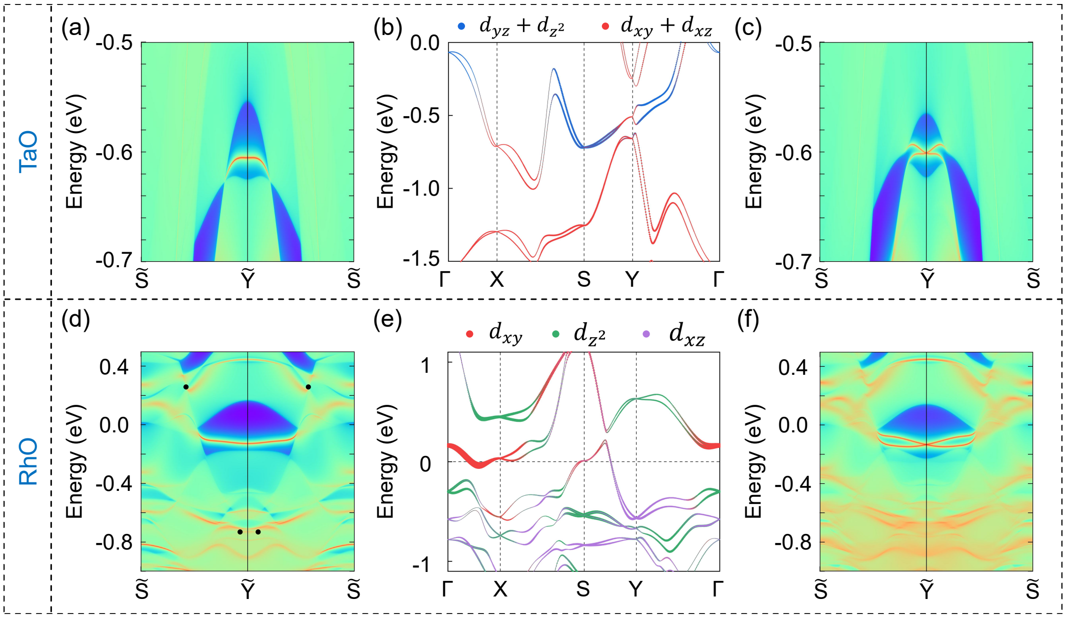

Figure S2: (a) and (c) Show the edge states of TaO without and with SOC, respectively, while (d) and (f) show those of RhO. (b) and (e) Present the orbital-projected band structures of TaO and RhO, respectively, with SOC included.

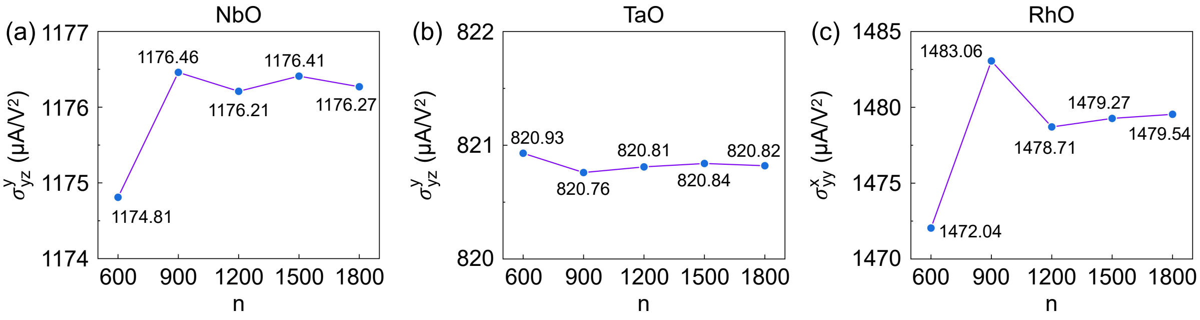

Figure S3: (a)-(c) Show the peak shift current as a function of k-mesh density (n×n×1) for NbO, TaO, and RhO, respectively.

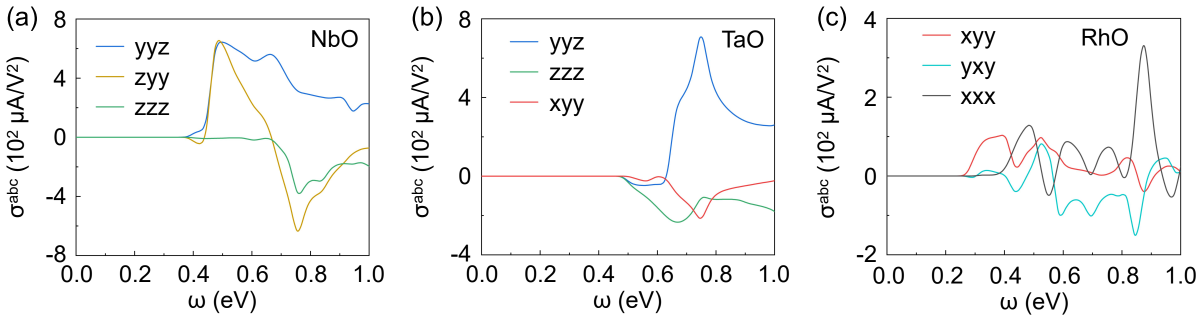

Figure S4: (a)-(c) Shift current spectra of NbO, TaO, and RhO, respectively, without SOC.

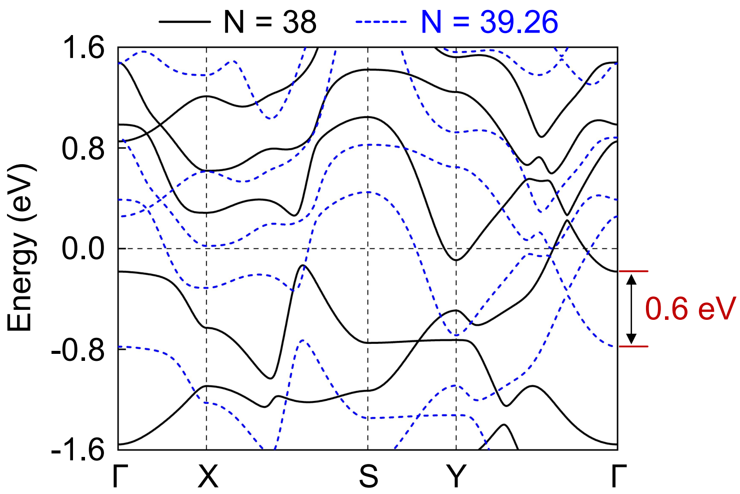

Figure S5: Comparison of band structures before and after electron doping.
